# Targeting Loss of Heterozygosity: A Novel Paradigm for Cancer Therapy

**DOI:** 10.3390/ph14010057

**Published:** 2021-01-13

**Authors:** Xiaonan Zhang, Tobias Sjöblom

**Affiliations:** Science for Life Laboratory, Department of Immunology, Genetics and Pathology, Uppsala University, SE-75185 Uppsala, Sweden; xiaonan.zhang@igp.uu.se

**Keywords:** loss of heterozygosity, cancer therapy, drug development and cancer evolution

## Abstract

Loss of heterozygosity (LOH) is a common genetic event in the development of cancer. In certain tumor types, LOH can affect more than 20% of the genome, entailing loss of allelic variation in thousands of genes. This reduction of heterozygosity creates genetic differences between tumor and normal cells, providing opportunities for development of novel cancer therapies. Here, we review and summarize (1) mutations associated with LOH on chromosomes which have been shown to be promising biomarkers of cancer risk or the prediction of clinical outcomes in certain types of tumors; (2) loci undergoing LOH that can be targeted for development of novel anticancer drugs as well as (3) LOH in tumors provides up-and-coming possibilities to understand the underlying mechanisms of cancer evolution and to discover novel cancer vulnerabilities which are worth a further investigation in the near future.

## 1. Introduction

**Figure 1:**
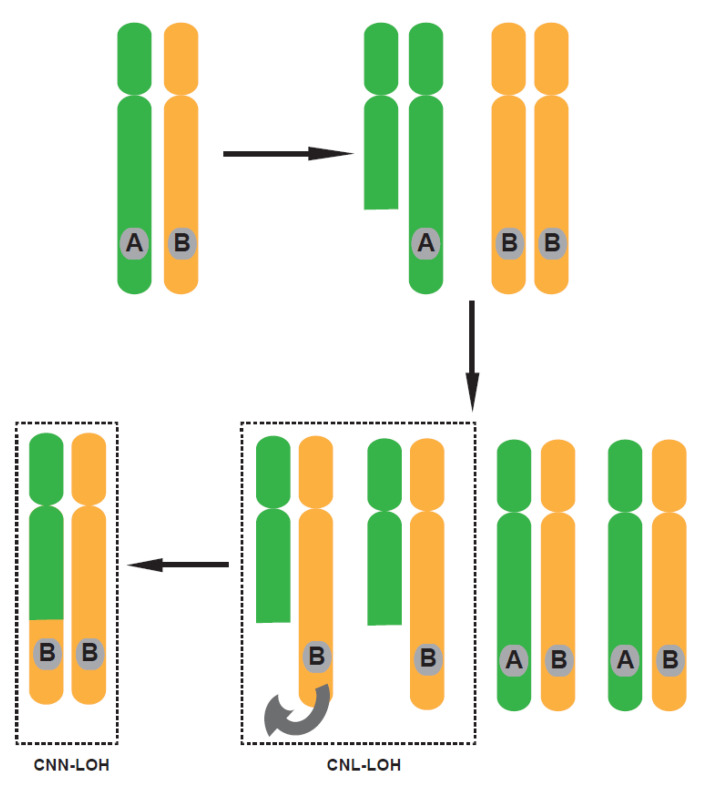
Two types of loss of heterozygosity. There are two main types of loss of heterozygosity (LOH), LOH with copy number losses (CNL-LOH) and copy number neutral LOH (CNN-LOH). Mitotic recombination is considered a major contributor to acquired LOH. During cancer progression, tumor cells may lose one allele (here referred to as A) as a consequence of partial chromosome deletion which is defined as LOH with copy number losses (CNL-LOH). CNL-LOH could further undergo recombination using the homolog (here refers as allele B) as a template for correction which is defined as LOH with copy number neutral (CNN-LOH). Here, orange and green regions represent either of two homologous chromosomes.

Several different somatic genetic and epigenetic processes contribute to the development of cancer, including copy number alterations, deletions, rearrangements or translocations of certain genes, somatic point mutations, and hypermethylation of promoters [1]. Loss of heterozygosity (LOH) was originally discovered using polymorphic markers which were heterozygous in germline DNA but homozygous in the tumor, and is common in the evolution of many cancer types [2]. In general, there are two types of LOH, (1) LOH with copy number losses (CNL-LOH), with a typical example of being losing the wildtype allele of a tumor suppressor, and (2) copy number neutral LOH (CNN-LOH), exemplified by the presence of two mutant alleles of *WT1* (11p), *FLT3* (13q), *CEBPA* (19q) and *RUNX1* (21q) which resulted in a growth advantage in tumors, such as in leukemia [3]. A complete or partial deletion of a chromosome leads to CNL-LOH, while CNN-LOH is mainly caused by acquired uniparental disomy (UPD) and gene conversion, and occurs without net change in the copy number [4,5] (Figure 1). In principle, the presence of two mutant alleles generated by CNN-LOH could lead to an alteration at the gene expression level, however, it has been recently shown that there are allelic differences in gene expression [6], indicating that the exact expression level could be associated with specific gene expression patterns and regulated by other mechanisms. This review will primarily focus on LOH with copy number loss (CNL-LOH), as it is a common phenomenon in cancer and more thoroughly investigated in comparison to copy number neutral LOH. The process of LOH is highly associated with a reduction of copy number of wild-type allele in individuals thus increases the impact of a genotype which becomes dominant when the function of wild-type allele is lost under LOH [4,7]. In certain types of cancers, such as colorectal carcinomas, LOH can affect more than 20% of the genome [8], causing loss of one allele of thousands of genes. Thus, LOH decreases the level of heterozygosity in cancer cells and creates significantly distinct genetic characters between tumor and normal cells.

## 2. Loss of Tumor Suppressor Genes by LOH

The function loss of tumor suppressor gene frequently involves the process of LOH, whereby a wild-type allele is lost, leaving only an inactivated allele in the cancer genome. A good example is the loss or inactivation of both alleles of the retinoblastoma gene (*Rb*) in retinoblastomas, which suggested that the *Rb* gene belongs to a class of human cancer genes which function as tumor suppressors [2,9]. Following this finding, numerous candidate tumor suppressors were discovered by characterizing sites of prevalent LOH in human cancers [10,11]. For example, as a consequence of LOH, the tumor suppressor gene *TP53* is inactivated, contributing to the development of many cancer types such as breast [12], lung and stomach [13] as well as chronic lymphocytic leukemia [14]. The LOH at 10q23, where the *PTEN* gene resides, has been linked to the development of breast cancer [15]. Further, there are inherited cancer predisposition syndromes which have germline mutations in tumor suppressor genes under LOH, such as *BRCA1* at 17q21. Women carrying germline LOH at *BRCA1* have an 85% lifetime risk of developing breast cancer and a greatly elevated risk of ovarian cancer [16,17]. *DPC4* (for deleted in pancreatic cancer, locus 4, *SMAD4*) on chromosome 18q was identified as a tumor suppressor gene in pancreatic, colon, bladder, biliary tumors as well as head and neck tumors [18,19,20]. LOH at the *DPC4* locus was detected in 51% of sporadic colon cancers [21]. Several more recently discovered tumor suppressor genes have also been reported to undergo LOH. For example, tripartite motif containing 3 (*TRIM3*) at 11p15.5 is lost in ~20% of glioblastomas (GBM) [22]. The Cut homeobox 1 (*CUX1*) gene at 7q22.1, suggested as both a potential tumor suppressor and an oncogene [23], is a target of LOH in many cancers. Taken together, loss of function of tumor suppressor genes frequently involves LOH and is linked to the development of cancer.

## 3. LOH Leads to Loss of Non-Driver Genes and Endows Cancer Cells with Unique Vulnerabilities

Beyond the direct consequences of LOH on bona fide cancer genes, particularly tumor suppressor genes, numerous non-driver genes located nearby or distally on the same chromosome arm may also undergo LOH. The establishment of LOH maps by cytogenetics [24] or, more recently, by genome-wide copy number analyses and genome sequencing, have enabled identification of chromosomal regions lost in different tumor types [25,26,27,28]. A study of ~10^5^ LOH events in 363 glioblastoma and 513 ovarian cancer samples revealed that LOH selectively occurs in early replicating regions, especially near RNA pol II-bound transcription start sites [29]. Together, these and other studies demonstrate that LOH in cancer genomes is not only affecting tumor suppressor genes but also numerous non-driver genes.

When one allele of an essential non-driver gene undergoes LOH in cancer cells, cancer cells should not be able to survive if the remaining allele is further lost or inhibited, whereas normal cells will be able to survive relying solely on the remaining allele [25], leading to an unique vulnerability in cancer cells. For example, several studies have together indicated that the process of LOH can affect the extent of ROS tolerance [30]. An LOH event relevant to ROS balance affects the *Oct1/Pou2f1* gene. The transcription factor Oct1/Pou2f1 is involved in a wide variety of functions in organism development, particularly relating to the development of neuroendocrine system [31]. Oct1 promotes glycolytic metabolism and mitotic stability and plays important roles in stress responses [32,33], and loss of one or both *Oct1* alleles has been associated with an upregulation of oxidative metabolism and increased levels of reactive oxygen species (ROS), thus inducing a coordinate metabolic shift and hypersensitive to oxidative stress [32,33]. Glutathione peroxidase (GPx-1) is a selenium-containing antioxidant protein mediating the reaction of hydrogen peroxide to water, using reducing equivalents from glutathione. A higher frequency of loss of heterozygosity at the *GPx-1* locus at 3p2 was first observed in lung tumors and lung tumor-derived cell lines [34]. Further studies revealed that *GPx-1* LOH is prevalent also in breast, colon, kidney and head and neck cancers [35]. Clinical investigations have revealed that LOH of non-driver genes could engender unique vulnerabilities in rare cancer types. Pheochromocytomas and paragangliomas are rare neuroendocrine tumors [36], and ~40% of tumors arise in patients with germline LOH of the succinate dehydrogenase (SDHx) genes. The SDHx protein is located on the inner membrane of mitochondria and plays an important role in cellular energy metabolism by linking the Krebs cycle to mitochondrial oxidative phosphorylation [37,38]. Taken together, loss of non-driver genes by LOH can cause unique vulnerabilities in cancer cells and thereby provide a novel class of therapeutic targets for cancer drug discovery and personalized medicine.

## 4. Loss of Heterozygosity Provides Novel Therapeutic Targets for Cancer Treatment

Targeted anti-cancer treatments typically rely on genetic differences between cancer and normal cells to achieve a specific inhibitory effect on cancer cells. Targets include oncogenes which are activated by mutation, tumor suppressor genes which are inactivated by mutation, or perturbed cell signaling in the maintenance of genome integrity or regular cellular metabolism [39,40]. However, the era of cancer genomics has revealed few novel targetable oncogenes but many tumor suppressor genes that, unfortunately, are difficult to target therapeutically [1,41]. For example, the TP53 tumor suppressor gene is mutated in >50% of human tumors [42]. Among the 393 amino acids of P53, sequences at positions 5–28 have p53 transcription activity and pathogenic mutations are enriched at R248Q, R273H, and R282W which affect DNA binding, or in R175H, Y220C, G245S, and R249S which are called conformational mutations [43,44]. The mechanism of p53 in cancer development has been thoroughly investigated, with more than 70K papers published since 1979. Nevertheless, there is still no drug targeting p53 available in the clinic, mainly due to the lack of a good binding site to serve as a direct target in the mutant p53 structure. Another example is *MYC*, which is amplified in nearly 14% of cases of at least 12 cancer types [45,46] and approximately 50% of patients with high-risk disease [47,48]. Currently, several MYC inhibitors have been identified from phenotypic screens, including 10058-F4, atorvastatin and the recently discovered Omomyc [49,50,51], however, all of them still have a long way to go from bench to clinic [52,53].

**Figure 2:**
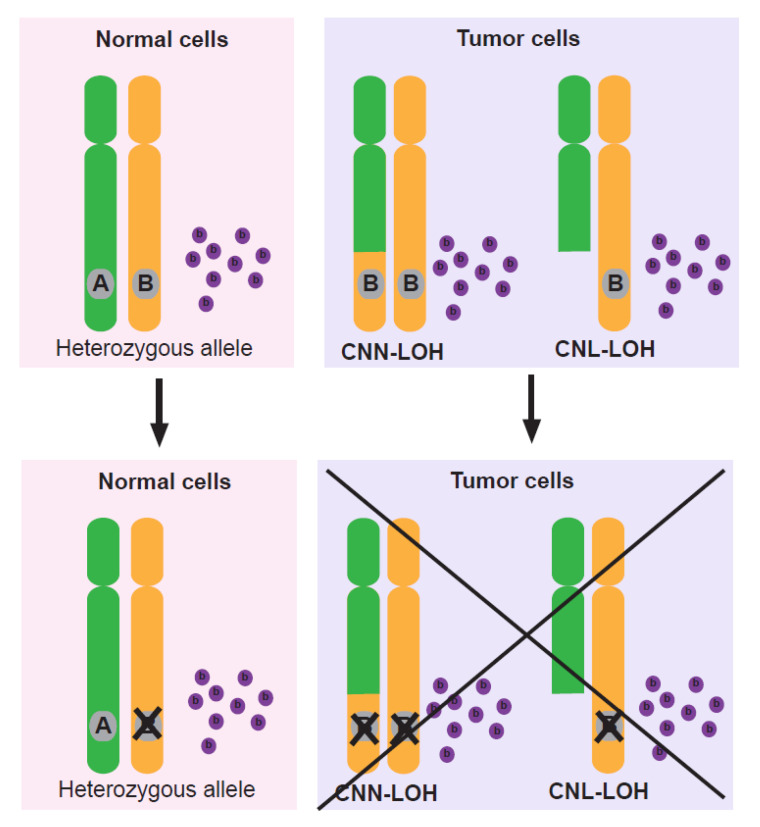
Targeting LOH in tumors for cancer treatment. When a cancer cell undergoes loss of heterozygosity of non-driver genes (A), further loss or inhibition of a specific allele (B) still retained in the tumor should not be tolerated, whereas normal cells will be able to survive relying solely on the retained allele. Treatment with an agent (purple dots) that is either a selective inhibitor of protein product B or a cytotoxic substrate metabolized by A but not B will result in selective killing of the tumor cells. Thus, approaches focusing on non-driver genes represent an alternative target class that merits further exploration. Here, orange and green regions represent either of two homologous chromosomes and ’X’ indicates the selective kill of cancer cells retaining only allele B.

Beyond the successful use of immunotherapy, it now appears necessary to go beyond the oncogenes and suppressor genes in the hunt for novel drug targets based on genomic aberrations. Thus, non-driver genes represent an alternative target class that merits further exploration (Figure 2). In 1999, the possibility of targeting LOH for anticancer therapy was first explored. The RPA70 gene at 17p13.3, which encodes the 70 kDa subunit of human replication protein A which plays a vital role in DNA replication, homologous recombination, and nucleotide excision repair in vitro [54] and any disturbance to the role of RPA70 is lethal to the cell [55]. RPA70 undergoes LOH in 44% of colon cancers, 58% of ovarian cancers, 20% of breast cancers, and 27% of non-small cell lung carcinomas. The oligonucleotide ISIS 1278 targeting segments of RPA70 mRNA containing variants effectively inhibited survival of cells expressing only the RPA70 mRNA with the exact complementary sequence, but was less effective in cells expressing the mismatched target, suggesting that developing anticancer agents based on normal genetic variation under LOH in essential genes is a feasible strategy for anticancer therapy [56].

Recently, the Beroukhim group have identified 5664 variants in 1278 essential genes that undergo LOH in cancer and pointed out that allele-specific inactivation of either of two essential genes (*PRIM1* and *EXOSC8*), which have been rigorously validated as genetic dependencies in cancer, is lethal in cancer cells [25,57,58]. *PRIM1* encodes the catalytic subunit of DNA primase and contains two common SNPs, of which one (rs2277339) undergoes frequent LOH across 33 different cancer types, occurring in 21% of lung adenocarcinomas, 18% of ovarian cancers, and 17% of pancreatic cancers. The other important gene reported in this study is *EXOSC8* encoding Rrp43, a component of the RNA exosome which is an essential multi-protein complex regulating RNA processing and degradation [58]. The position of rs117135638 in *EXOSC8* undergoes LOH in 29% of cancers, including 72% of lung squamous cell carcinomas, 62% of ovarian cancers, 46% of lung adenocarcinomas, and 40% of breast cancers. Allele-specific disruption using sgRNA targeting rs2277339 in *PRIM1* and rs117135638 in *EXOSC8* strongly reduces the growth in cells containing targeted LOH allele of rs2277339 or rs117135638, while cells harboring the non-targeted allele remain intact [25]. Although there were no followed drug screens by targeting the products of the two essential genes, the authors discussed the possibility of developing allele-specific small molecule inhibitors using the canSAR protein annotation tool [59,60] which provides a prioritization of targets based on general drugability, suggesting that cancer vulnerabilities generating from LOH represent viable targets for novel anticancer drug development.

We recently demonstrated that cancer cells will develop unique vulnerabilities when LOH events affect non-essential genes. We analyzed variants obtained from 1092 clinical samples in the 1000 Genomes project and selected 23,532 non-synonymous small nucleotide variants (SNVs) in functional protein domains with LOH frequency ≥0.5% for a further confirmation and 45 common non-synonymous small nucleotide variants (nsSNVs) near the catalytic sites of 17 enzymes that frequently undergo LOH were identified. After a series of strict filtration and selection, the gastrointestinal drug metabolic enzyme N-acetyltransferase 2 (*NAT2*) at 8p22, which is frequently lost in colorectal cancers, was confirmed as a top target. It is worth noting that, the following proof of concept experiments suggested that NAT2 harboring mutant allele (NAT2*6A, rs1799930) has a 10-fold reduced activity comparing with the wild type allele (NAT2*13A). From a total of 189,018 compounds, we identified 6-(4-aminophenyl)-*N*-(3,4,5-trimethoxyphenyl) pyrazin-2-amine (APA) which preferentially kills cells expressing slow NAT2 (NAT2*6A, rs1799930) [26]. Comparing to the previously described efforts, this represented the first compound targeting cancer vulnerabilities stemming from LOH of non-cancer genes which supports that LOH targeting is a practically exploitable paradigm in the discovery of novel drugs for cancer treatments. 

It is noteworthy that while only one or two target genes were selected for further validation in these studies [25,26], several other loci were reported within each respective study. These are no doubt rich resources for pursuing novel drug targets among the cancer vulnerabilities associated with LOH.

## 5. Loci Undergoing Loss of Heterozygosity as Clinical Biomarkers

Loss of heterozygosity is a common genetic event in the development of many cancer types and occurs in every step of tumorigenesis [2]. At the same time, several studies have demonstrated that LOH at a specific gene locus could be used as biomarkers of cancer risk or the prediction of clinical outcomes in certain types of tumors. Here we summarized a subset of reported LOH related biomarkers (Table 1). Some have been extensively studied, such as the tumor suppressor *TP53* on 17p13, while others are relevant for certain tumor types, such as *HLA-A02* in patients with synovial sarcoma.

### 5.1. Biomarkers on Chromosome 1q Associated with LOH

In primary neuroblastoma, loss of heterozygosity on 1p36 has been reported in 23–35% of patients, and was shown to be significantly associated with prognostic markers of aggressive neuroblastoma when patients are diagnosed. Moreover, loss of 1p36 has been reported to predict both poor event-free and overall survival [61,62] and the genomic status of 1p has been implemented as a risk stratifying marker in the German trial NB2004 [83]. The underlying driver gene mutation is unknown, but may involve one or more neuroblastoma tumor suppressor genes and loss of 1p36 could promote tumor growth and favor the effects from the *MYCN* oncogene [84,85].

### 5.2. Biomarkers on Chromosome 3q Associated with LOH

*FRA3B* locating on chromosome 3p14.2, is one of the most active common fragile sites in the human genome and has been reported to incur deletions or translocation breakpoints in many types of cancers including lung cancers [86]. The *FHIT* gene is one of the most well-known genes at this common fragile site and has been reported to undergo LOH in >76% of lung cancer [87,88]. Interestingly, several studies have revealed that a significantly higher level of *FHIT* LOH occurs in lung cancers from smokers compared with that from nonsmokers, indicating that this could be a predictive marker of an early event in the genesis of smoking-related cancers [65,66]. Another example is *CACNA2D3* encoding a protein which is an auxiliary member of the alpha-2-delta subunit family of the voltage-dependent calcium channel complex. There are two commonly SNP sites undergoing LOH (rs589281, rs6797113) at 3p21 which have been reported to associate with poor clinical outcome in esophageal cancer [67,68]. The von Hippel-Lindau (VHL) tumor suppressor gene at 3p25 plays a vital role in the regulation of other genes and control of cell division. LOH at two loci (rs1642742 and rs1642743) of VHL gene results in a loss of VHL protein function, has been proposed as a candidate predictive biomarker for clinical outcome in clear-cell renal-cell carcinoma (ccRCC) patients [69].

### 5.3. Biomarkers on Chromosome 6q Associated with LOH

Loss of heterozygosity of the human leukocyte antigen (HLA) locus locating on Chromosome 6p21 was associated with low peptide diversity in colorectal cancers (CRCs), entailing a poor response to immune checkpoint inhibitors such as inhibitors targeting mitogen-activated protein kinase kinase (MEK) [70]. Similarly, LOH at *HLA-A02* has been proposed as a predictive biomarker for synovial sarcoma and is prognostic of poor outcome [71]. Fatty acid-binding protein-7 (FABP7) is involved in the transportation of intracellular long-chain fatty acid [89] and has been shown to regulate several pathways leading to the inhibition of cell proliferation and tissue differentiation [90]. The expression of FABP7 correlated with survival rate in patients with glioblastoma [72] and the frequency of LOH at 6q22.31 was 50% in metastatic melanomas, compared with 0 of 14 in primary melanomas (*p* = 0.0017) indicating that FABP7 is a potential diagnostic biomarker of early-stage melanoma dissemination in blood [73].

### 5.4. Biomarkers on Chromosome 16q Associated with LOH

The B-lymphocyte antigen CD19 at 16p11.2 is expressed in all B lineage cells [91]. The chimeric antigen receptor (CAR) T cell therapy CTL019 (Tisagenlecleucel) is designed to use T cells of patients expressing a CD19-specific, 4-1BB/CD3 ζ-signaling CAR [92]. Patients with relapsed or refractory B cell acute lymphoblastic leukemia (B-ALL) receiving CTL019 have achieved 70% to 94% complete remission in several clinical trials. However, more than 30% of tumors that responded to CTL019 therapy eventually recurred [93,94] which was found to be caused by frameshift mutations leading to a truncated protein with a nonfunctional or absent transmembrane domain and consequently to a loss of surface antigen [77]. Notably, there was no *CD19* LOH in samples from 7 untreated patients), however, 8/9 relapsed patients had acquired LOH at *CD19* [95], indicating that irreversible loss of heterozygosity in *CD19* could be used as a biomarker for an outcome prediction after the CAR T cells therapy CTL019 [77].

### 5.5. Biomarkers on Chromosome 17q Associated with LOH

The tumor suppressor TP53, located on 17p13.1, contains 11 exons of which the genomic integrity of exons 5-8 plays a particularly important role for its activity. These *TP53* gene exons are also mutational hotspots in cancer [96,97] and >80% of *TP53* mutations occur within this region. So far, more than 27,000 somatic mutation in *TP53* have been catalogued in the International agency for research on cancer (IARC) *TP53* database [98]. Further, LOH at 17p13.1 is one of the most frequent genetic alterations leading to human cancers [78,79]. Analysis of *TP53* mutations from 1,420 tumor samples revealed that loss of one *TP53* allele may be a sufficient driver in breast cancers of the luminal B subtype as a higher fraction of wild-type tumors with LOH is noticed [99]. Codon 72 of the *TP53* gene contains a well-known polymorphic site with the two alleles arginine and proline (Arg72Pro) [100]. Analysis of *TP53* mutations from 204 Danish women and revealed that heterozygous patients losing either the Arg72 or Pro72 variant in the *TP53* gene because of LOH had a reduction in disease-free survival compared with patients retaining the polymorphism (Arg72Pro) [101]. Another analysis revealed that patients with homozygous deletion under LOH or both del17p and *TP53* mutation had a significantly worse outcome, comparing with patients who had only del17p or *TP53* mutation (3-year overall survival 84% versus 29% (*p* = 0.02) and 3-year progression-free survival 73% versus 29% (*p* = 0.04), respectively) [102].

### 5.6. Biomarkers on Other Chromosomes Associated with LOH

Loss of heterozygosity at 9p was observed to be significantly associated with poorer prognosis of glioma patients [74]. Another study reported that LOH at 9p13 was associated with poor survival in squamous cell carcinoma/adenocarcinoma patients who had surgical resection. The nel-like1 *(NELL1*) gene at 11p15 is reported to frequently undergo loss of heterozygosity in esophageal adenocarcinoma (EAC) [76]. Another study from Bepler et al. reported an association between *NELL1* LOH at position of 11p15.5 and poor survival outcome in 180 patients who had lung cancer [103]. A number of studies have revealed that colorectal cancer patients at stage III with LOH at *SMAD4* at 18q showed a poorer overall 5-year survival rate comparing with patients harboring non-18q LOH [81]. A meta-analysis of data from 27 studies and 2189 patients [82] extended this finding, showing that LOH on chromosome 18q could assist in predicting the clinical outcome after therapies in stage III colorectal cancer (CRC).

## 6. Loss of Heterozygosity in Tumors Provides Up-and-Coming Possibilities to Understand the Underlying Mechanisms of Cancer Evolution

As described above loci undergoing loss of heterozygosity could be used as clinical biomarkers or the prediction of clinical outcomes in certain types of tumors and provide novel therapeutic targets for cancer treatment which have been proved applicable in current different studies. Here, last but not the least, studies of loss of heterozygosity in tumors also provides up-and-coming possibilities to understand the underlying mechanism of cancer evolution which surpasses the scheme of targeting point mutations in tumorigenesis and focuses on the contribution of losses and gains of genetic material through whole chromosomes or chromosome arms [1].

A very recent study [104] relating to non-small cell lung cancer (NSCLC) has observed a significant enrichment for whole-genome doubling (WGD) which is coordinated with an extensive loss of heterozygosity, indicating that loss of heterozygosity and whole-genome doubling are common events [105]. Then they pursue to understand the effects of natural selection on LOH and WGD during cancer evolution by comparing the early (pre-WGD) and late (post-WGD) mutations within segments of LOH based on their modified dN/dS ratio. The dN/dS ratio has a long history in the study of selection in species evolution [106,107], by comparing synonymous (silent; dS) and nonsynonymous (amino acid-changing; dN) substitution rates in protein-coding DNA sequences [107]. Based on their modified dN/dS ratio test [41] and a well-accepted criteria: ratio of dN/dS > 1 indicates a positive selection and an enrichment of non-silent mutations in cancer genes [41,108,109], López et al. [104]. focused on identifying new cancer genes based on the dN/dS ratio which plays an important role in cancer evolution. Firstly, using their method for calculating dN/dS ratio and a series of strict filtration, the two well-established tumor suppressor genes *TP53* and *PTEN* were discovered with a dN/dS ratio > 50 in lung squamous cell carcinoma (LUSC), indicating the reliability of their analysis method. *ZNF750* (zinc finger protein 750) which is described as a tumor suppressor gene in squamous cell carcinoma [110,111], is as well subject to high positive selection (dN/dS ratio > 50) in regions of LOH in lung squamous cell carcinoma (LUSC) and head and neck squamous cell carcinoma (HNSC) [104]. Notably, they further expanded the analysis to the remaining cancer subtypes in TCGA and 33 potential tumor suppressor genes were identified by limiting the analyses to sections of LOH and 14 additional novel genes (*WWC1, FPF12, NT5DC3,NCLN, KRTAP195, GRIK2, GLRA1, FAXDC2, FAM19A3, CRYGC, CLEC4E, BC02, ARPP21* and *AC061992.1*) were discovered which were assumed tumor suppressor genes but not included in the catalogue of somatic mutations in cancer (COSMIC) [112]. This approach provides a unique angle to discover novel cancer genes which play roles in cancer evolution. Moreover, many following studies could be carried out based on this discovery, such as cancer prediction and targeted cancer therapies.

Recently, Zaccaria et al. [113] introduced another algorithm named copy-number haplotype inference in single cells using evolutionary links (CHISEL) to study cancer genes which are involved in the early stage of tumor evolution. CHISEL algorithm is able to deduce allele- and haplotype-specific copy numbers in single cells [113] and overcome the problems in already existing single-cell barcoding technologies which are usually caused by the extremely low sequencing coverage (<0.05× per cell). Based on CHISEL algorithm, they reanalyzed 10× genomics chromium single-cell DNA sequencing data from two patients at early stage of breast cancer (patient 1: a triple negative ductal carcinoma in situ and patient 2: at stage 1 infiltrating ductal carcinoma) and identified large-scale allele-specific copy-number aberrations (CNAs) which, on average, cover ~25% of the genome in at least 100 cells however were uncharacterized by previous used total copy-number analysis. Further investigation into the ~25% CNAs of the genome revealed that the most common type of allele-specific CNAs identified by CHISEL are copy-neutral LOHs and contains several genes which have been reported in the previous 560 breast cancer database [114], such as *ARID1B* and *ESR1* on chromosome 6q, *PTEN* on chromosome 10q, *BRCA2* and *RB1* on chromosome 13, It is worth noting that all the genes undergoing copy-neutral LOHs have been reported in almost all type of tumor cells [115,116,117,118,119,120,121,122,123,124,125,126,127], indicating that these copy-neutral LOHs mutations may take an important role at an early stage during the tumor evolution. 

In 2018, Palin et al. [128] have performed a high-resolution allelic imbalance (AI) landscape in 1699 colorectal tumor/normal DNA pairs and among them, 256 were later whole-genome sequenced. The results revealed that three loci commonly undergoing LOH are *TP53* (63%, 1072 of 1699 cancers), *SMAD4* (61% or 1040 of 1699 cancer) and 8p21.3 (47% or 806 of 1699 cancers) [128]. It is worth noting that 8p21.3 shows no obvious candidate target genes. However, previous studies have revealed that a frequent loss of chromosome 8p is firmly linked to colorectal tumorigenesis [129,130] and 8p loss and *MYC* gain (also in 8q) significantly co-occurred in the same samples, indicating that a frequent loss of 8p may at least to some extent be an accompanying event which is driven by the strong positive selective of *MYC* gain.

Moreover, the study from Palin et al. is as well important to understand the mechanism underlying cancer evolution. When discussing tumorigenesis, we always like to refer to the hallmarks of cancer [131], believing that cancer is the process of accumulating mutations of genes. However, one common question based on this theory is still hard to answer: why so few genes are commonly mutated in most cancers [1,132] and one obstacle occurs in cancer therapies which is still hard to solve: commonly mutated genes such as *TP53* [1,41] and MYC [52,53] are hard to target. This study of developing genetic aberrations provides a fresh perspective that it is not point mutations which are frequently selected for tumorigenesis. Instead, gross chromosomal changes which could affect the expression levels of cancer genes as well as many other genes through a single event during the process of tumorigenesis. Although these studies mainly focused on colorectal cancer [128,133,134,135], the conclusion encourages researchers to carry out similar studies in other types of cancer. It is believed that studies of loss of heterozygosity in tumors provide up-and-coming possibilities to understand the progress of tumorigenesis which is a totally different but complementary aspect to studies of targeting point mutations.

Moreover, emerging studies have shown that findings on loss of heterozygosity shed a light on understanding the mechanism underlying tumor immune evasion during tumor evolution. An important step in the process of neoantigen presentation and cytolytic T cell response is regulated by class I human leukocyte antigen (HLA), which enables recognition by T cell receptors through expressing intra-cellular peptides on the surface of cell [136,137]. Thus, the downregulation of HLA expression at transcriptional or translational level should be able to reduce the ability of antigen presentation, leading to the consequence of immune evasion. Indeed, the downregulation of HLA presentation has been discovered in several individual studies and has as well been shown to link to poor outcome in clinical therapies across different cancer types [138,139,140,141,142]. The first report trying to explain this phenomenon from the perspective of loci undergoing loss of heterozygosity was published in 2016 [143] and described that after the treatment of seven patients with lung metastases by infusing ~1.11 × 10^11^ HLA-C*08:02—restricted tumor-infiltrating lymphocytes, six out of seven patients observed an objective regression on evaluation after 9-month therapy. However, one patient had progressed and the sequencing result revealed that this patient has lost the chromosome 6 haplotype encoding the HLA-C*08:02 class I major histocompatibility complex (MHC) molecule, providing a mechanism for tumor immune evasion [143]. A similar result revealing that loss of heterozygosity of the human leukocyte antigen is associated with low peptide diversity and leads to a poor response to immune checkpoint inhibitors has been reported in colorectal cancers [70]. Another study relating to lung cancer accelerated the study about the relationship of loss of heterozygosity at HLA and immune escape in evolution of lung cancer [144]. After analyzing the HLA locus in 288 TRACERx non-small-cell lung cancer (NSCLC) exomes from 96 patients [145,146], researchers observed that LOH occurs in 40% of early-stage non-small-cell lung cancers, indicating that LOH HLA could be an advantage during the evolution of non-small-cell lung cancers. To clarify the timing of HLA LOH in NSCLC evolution, sequencing data from 37 NSCLC primary tumors with matched brain metastases were analyzed [147], revealing that 46% had HLA LOH with a trend toward enrichment of HLA LOH in brain metastases from the same patient, comparing with the primary tumor originally from lung. Thus, LOH HLA more likely appears at a later stage in cancer development and may provide an advantage under natural selection in order to escape immune predation. These findings further illustrated the importance of LOH in cancer evolution and tumor immune escape. As more pre- and post-therapy data emerges from different cohorts, there is great potential to investigate the extent of HLA LOH in different cancer types at different stages of cancer evolution, to improve understanding of the mechanisms underlying tumor immune evasion. Following the idea in this study, the other immune related LOH loci would be discovered in other research.

## 7. Future Perspective

Loss of heterozygosity (LOH) is a common genetic event in the development of cancer and is known to play an essential role in the somatic loss of wild-type alleles in cancers. Moreover, a vast number of non-driver genes concomitantly undergo LOH, corresponding to more than 20% of the total genome, such as in colorectal carcinomas [8,148]. This creates patient specific differences between tumor and normal cells, which may in turn generate vulnerabilities in cancer cells that could potentially be targeted. Here, we have reviewed and summarized mutations associated with LOH on chromosomes which have been shown to be promising biomarkers of cancer risk or predictive of clinical outcomes in certain types of tumors. Moreover, with the development of genome-wide analysis of clinically relevant data, targeting cancer cell vulnerabilities associated with LOH could provide a novel paradigm for cancer therapies in populations harboring the same LOH allele. As loss of heterozygosity is a common genetic event in cancer genomes, analysis of LOH could in addition provide a therapeutic option for precision medicine. Importantly, LOH specific inhibitory probes or cytotoxic drugs from small molecule screening have been identified, proving the concept that LOH targeting in cancer cells is a fertile avenue for novel anticancer drug discovery and development [26]. To progress this field, additional genomic analysis based on data from large clinical sample cohorts should be performed in order to find exploitable nucleotide level and structural variants at LOH loci in common cancers. Functional studies should be performed to validate druggable non-driver genes which are either essential for cell survival, proliferation or involved in vital metabolic cellular activities. Last but not the least, we have addressed several up-to-date findings relating to the importance of LOH for understanding the mechanisms underlying the progress of cancer evolution [104,128,149]. Methods of analyzing genomic data are continuously improving, and some cancer genes which have not been found based on previous methods have yet been discovered in recent work [104] and some basic questions which have not been fully addressed have currently been partially settled by a high-resolution allelic imbalance landscape [128]. Thus, studies relating to the LOH in cancer cells will help to further understand differences between tumor and normal cells as well the mechanism underlying cancer evolution and tumor immune evasion, opening novel avenues for cancer therapy development. At the same time, there is a highly need to utilize the results/findings from these studies, such as perform necessary investigations to look into the biological functions of the LOH genes among the top list [104,113] or novel strategies to target proteins which are translated from a gene harboring LOH loci [26,142,144,150].

## Figures and Tables

**Table 1:** Biomarkers on Chromosomes associated with LOH.

Chromosome Associated with LOH	Gene Name	LOH Position	Predictive Biomarker	Reference
1p	N/A	1p36	Significantly associated with prognostic markers of aggressive neuroblastoma when patients are diagnosed	[61,62,63,64]
3p	*FRA3B*	3p13–3p21	Predicted early event in the genesis of smoking-related cancers.	[65,66]
	*CACNA2D3*	3p21	Associated with poor clinical outcome in esophageal cancer.	[67,68]
	*VHL*	3p25	Predictive biomarker for clinical outcome in clear-cell renal-cell carcinoma (ccRCC) patients.	[69]
6q	*HLA*	6p21	Leads to a poor response to immune checkpoint inhibitors.	[70]
			Predictive biomarker for patients with synovial sarcoma and is prognostic of poor clinical outcome.	[71]
	*FABP7*	6q22	Correlated with survival in patients with glioblastoma.	[72]
			A potential diagnostic biomarker of early-stage melanoma systemic spreading in blood.	[73]
9q	N/A	9p13	Significantly associated with poorer prognosis of glioma patients.	[74]
10q	*PTEN*	10q23	Functionally related to the development of breast cancer, associated with poor prognosis	[75]
11q	*NELL1*	11p15	An association between LOH at 11p15.5 and poor survival in 180 lung cancer patients.	[76]
16q	*CD19*	16p11	Irreversible loss of heterozygosity in CD19 could be used as a biomarker for an outcome prediction after the CAR T cells therapy CTL019.	[77]
17q	*TP53*	17p13	One of the most frequent genetic alterations leading to human cancers.	[78,79]
	*BRCA1*	17q21	A germline LOH on *BRCA1* confront an 85% lifetime risk of breast cancer and a greatly elevated risk of ovarian cancer	[80]
18q	*DPC4 (SMAD4)*	18q21	Assist in predicting the clinical outcome after therapies in colorectal cancer (CRC) patients.	[81,82]

## Data Availability

Not applicable.
